# Assessment of biodegradation in ancient archaeological wood from the Middle Cemetery at Abydos, Egypt

**DOI:** 10.1371/journal.pone.0213753

**Published:** 2019-03-27

**Authors:** Ahmed M. Abdel-Azeem, Benjamin W. Held, Janet E. Richards, Suzanne L. Davis, Robert A. Blanchette

**Affiliations:** 1 Botany Department, Faculty of Science, Suez Canal University, Ismailia, Egypt; 2 Department of Plant Pathology, University of Minnesota, St. Paul, Minnesota, United States of America; 3 Director of Abydos Middle Cemetery Project, Department of Near Eastern Studies, University of Michigan, Ann Arbor, Michigan, United States of America; 4 Conservator, Abydos Middle Cemetery Project, Kelsey Museum of Archaeology, University of Michigan, Ann Arbor, Michigan, United States of America; Universita degli Studi di Milano, ITALY

## Abstract

Abydos is a large, complex archaeological site located approximately 500 km south of Cairo in Upper Egypt. The site has served as a cemetery for thousands of years and is where most of the Early Dynastic royal tombs are located. North Abydos includes the Middle Cemetery and the North Cemetery, which are separated from each other by a wadi. The Middle Cemetery was the burial ground for important Sixth Dynasty (2407–2260 BC) officials and over time for thousands of elite and non-elite individuals as well. Excavations at the core area of the Old Kingdom mortuary landscape have revealed many culturally important wooden objects but these are often found with extensive deterioration that can compromise their preservation. The objectives of this study were to characterize the biodegradation that has taken place in excavated wooden objects, elucidate the type of wood degradation present, obtain information on soil properties at the site and identify fungi currently associated with the wood and soils. Light and scanning electron microscopy studies were used to observe the micromorphological characteristics of the wood, and culturing on different media was done to isolate fungi. Identification of the fungi was done by examining morphological characteristics and extracting rDNA from pure cultures and sequencing the ITS region. Wooden objects, made from *Cedrus*, *Juniperus* and *Acacia* as well as several unidentified hardwoods, were found with extensive degradation and were exceedingly fragile. Termite damage was evident and frass from the subterranean termites along with sand particles were present in most woods. Evidence of soft rot attack was found in sections of wood that remained. Fungi isolated from wood and soils were identified as species of *Aspergillus*, *Chaetomium*, *Cladosporium*, *Fusarium*, *Penicillium*, *Stemphylium Talaromyces* and *Trichoderma*. Results provide important information on the current condition of the wood and gives insights to the identity of the fungi in wood and soils at the site. These results provide needed information to help develop conservation plans to preserve these degraded and fragile wooden objects.

## Introduction

**Fig 1:**
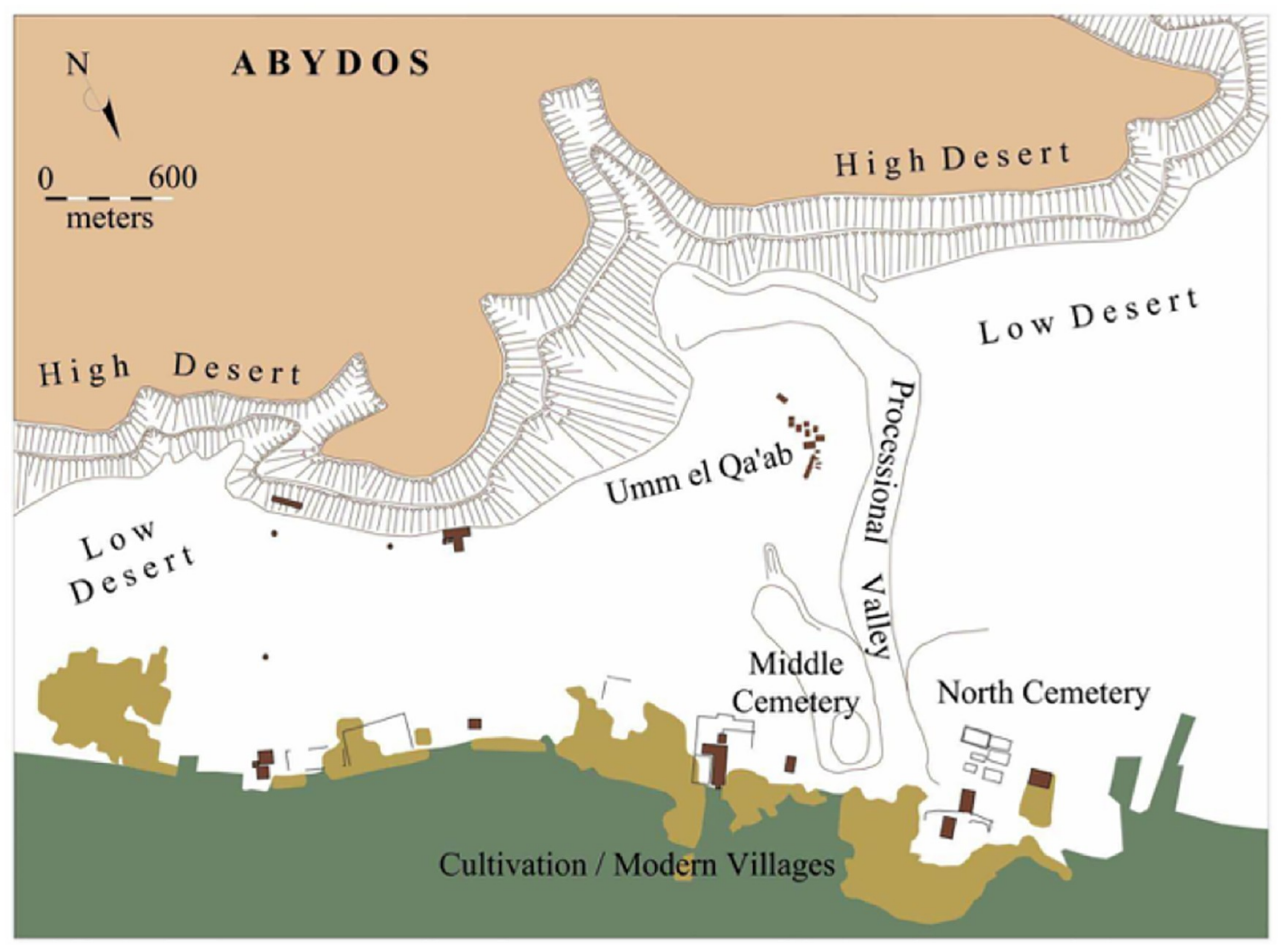
Map showing the Middle Cemetery at Abydos (J. Richards after a base map used with permission of Matthew D. Adams).

Abydos is a large archaeological site located approximately 500 km south of Cairo in Sohag Governorate, near El-Balyana City in southern Egypt. The cemetery is located on the low desert plateau of the Western Desert, adjacent to the village of Beni Mansour at the edge of the flood plain. Abydos is one of the most important archaeological sites in Egypt since it was the burial place of the first pharaohs of the united Egyptian state and the center of the cult for the god of the dead, Osiris [1]. The site is complex, consisting of cemeteries, settlements, and ritual spaces, divided into two main sections: South and North Abydos (Fig 1). The low desert landscape of North Abydos is, in turn, divided into the Middle Cemetery and the North Cemetery, separated from each other by a wadi which leads to the area known as Umm el-Qa’ab, where the Early Dynastic royal tombs are located [2]; and the area of Kom el Sutan, the location of the ancient town and Osiris temple. While mortuary activity throughout this landscape was initially restricted in the wake of its use for royal burials at Umm el-Qa’ab, the Middle Cemetery was subsequently primarily used for private funerary activity beginning in the later Old Kingdom including the burial sites for important officials of the regional and central government, e.g., the mastabas of Weni the Elder, Governor of Upper Egypt, his father the Vizier Iuu, and other individuals [3–7].

A number of studies have been carried out to document and characterize wood decay found in historical and archeological wooden structures in terrestrial sites [8–15]. These investigations have provided information on the types of wood decay present in different environments and the various processes of degradation that may occur. Micromorphological observations have also been found to provide valuable information on the type of fungi causing the attack since decay signatures are different among different fungi. There are, however, very few reports providing information on decay in archeological wood from Egypt. A few previous studies have showed both abiotic deterioration and biotic forms of wood decay in museum collections of archaeological wood from Egypt [16–18]. Other investigations in Egypt reporting wood decay in trees or historical buildings are very limited and focus on decomposition processes under laboratory conditions [19–24]. Greater knowledge of the fungi involved in wood decomposition processes has also been obtained with advances in molecular techniques that have made it possible to accurately identify fungi by sequencing conserved regions of rDNA [25–27].

Archaeological wood found with advanced decay is fragile due to the effects of degradation that can cause severe losses in wood strength and other physical properties. The wood found at this Old Kingdom burial site represents some of the world’s oldest wooden objects and they are exceedingly important since they provide a great deal of important information about the culture of ancient Egypt. Successful conservation is essential if these remarkable objects are to be preserved for study and display. Knowledge of the current condition of the wood and changes that have taken place in the woody cell wall structure facilitates the selection of appropriate methods for consolidation and treatment.

The objectives of this study were to investigate recently excavated wood from the Middle Cemetery at Abydos and characterize the biodegradation occurring, elucidate the type of wood degradation present in the wooden objects, obtain information on soil properties adjacent to where wood was excavated and identify the fungi isolated from soil and wood at the site by sequencing the ITS region of rDNA.

## Materials and methods

### Archaeological wood samples examined

Thirty-five wood samples were studied from a number of recently excavated wooden objects (Table 1). Small segments of the degraded wood or fragments associated with an object were taken (excised from fragments associated with intact objects). Sections were made to reveal the wood just beneath the surface and observed microscopically at Abydos and also placed in culture media. Wood anatomical characteristics of sections made at the site were used to identify the woods although the minute size of some samples was not sufficient to identify some of the hardwood samples. This work was carried out under the research permit granted by the Permanent Committee of the Ministry of Antiquities, Egypt to the University of Michigan and Janet Richards as project director and decay assessment under permit to Robert Blanchette from the Egypt Director General of the Secretariat of the Council of Museums Sector Management, Supreme Council of Antiquities. All necessary permits were obtained for the described study, which complied with all relevant regulations.

**Table 1:** List of wood samples examined for this study. Samples are accessioned in the official Ministry of Antiquities Storage Magazine at the site of Abydos, Egypt.

Sample Number	Object Identification Number	Type of Object
1	AMC01 Unit 9 Weni	wood fragment tomb of Weni
2	AMC09 Level 25 (March) Serdab	small statue / wood model
3	AMC07 Op 12 Level 02a	miscellaneous wood fragment
4	AMC13 Unit 26 Level 11 (1 April)	coffin
5	AMC01 level 1 Unit 9 Weni tomb	lid of a wood box
6	AMC99 Weni tomb	side of box
7	AMC13 Unit 25 Level 8	coffin
8	4th April (Site Coffin Frag.)	coffin
9	AMC2013 Level 7 Op 25	coffin
10	AMC01 Level 18	coffin base unit and joint
11	AMC2013 3.4	coffin
12	AMC07, OP 12, Level 2b	miscellaneous wood fragment
13	Coffin in site 7	coffin
14	AMC2013 Field Coffin Sample 7	coffin
15	AMC01 OP7	small coffin
16	AMC1999, Unit 07E, Level 2	base of wood statue
17	AMC01 Level 1 Weni tomb	outer coffin
18	AMC2013 Op 25 Level 7 25 March	coffin
19	AMC01 Op 07 Level 5	coffin
20	Coffin at Site 4 April 2013	coffin
21	AMC2009 Level 25 Serdab	small statue / wood model
22	AMC2013 Level 3 Coffin Wood	coffin
23	AMC2013 Coffin at Site	coffin
24	AMC2013, Level 14, Op 25	miscellaneous dowel fragment
25	AMC07, Unit 10, Level 3	miscellaneous fragment
26	AMC09 Level 25 Serdab	statue / wood model
27	AMC07, Iuu Ka Statue	base of statue
28	AMC2013, Op 25, level 7	coffin
29	AMC2013, Unit 26, Feat. 26.9	coffin
30	AMC2013, Unit 28, Feat. 14, Burial 26.4	coffin
31	AMC2013 Op 25, Level 7, March 25	coffin
32	AMC2013 Op 25, Level 7	coffin
33	AMC 2013, Baby Coffin	small coffin
34	AMC2013, Lower Coffin at Site, April 3	coffin
35	AMC2007, Unit 11, Feat. 30 level 59	coffin

### Isolations from soil and wood

Twenty-five soil samples (five per site) were also collected below the surface at five sites in close proximity to where coffin samples outside the tomb had been excavated. The coordinates for these collections were approximately N26.1115 E31.5441. Soil samples were transferred to the laboratory in sterile polyethylene bags. The five soil samples from each location were mixed to make a composite soil sample for each site. The composite soil samples were air dried and passed manually through a 2-mm sieve before analyses. The pH values of water extracts of soil samples and total soluble salts as electrical conductivity (micro-Siemens/cm) were determined with a pH meter (model 201, Orion research Co.) and conductivity meter (Corning 311) respectively using a water to soil ratio of 2:1 [28]. Organic matter content was determined by loss-on-ignition, where loss is calculated as a percent of the oven-dried sample [29]. The mechanical analysis and classification of soil samples were carried out using method as described by United States Department of Agriculture [30].

Five composite soil samples (five from each location) were used for culturing and a dilution plate method was used for isolations [31]. Soil and wood samples were plated on several types of media including Czapek yeast extract agar (CDH Fine Chemicals), potato dextrose agar (Difco), and malt extract agar (Difco) according to the manufacturer’s directions. In addition, an acidified malt extract agar (with 2 ml concentrated lactic acid added after autoclaving) and a selective culture medium for isolating basidiomycetes (15 g malt extract, 15 g agar, 2 g yeast extract, 0.06 g benlate (Methyl-L-(butylcarbamoil)-2-bencimidazol-carbamate) with 2 ml concentrated lactic acid, and 0.01 g streptomycin sulfate added after autoclaving. For each type of media, 10 plates were used for recovering soil fungi from each site. Incubation was at room temperature (28± 2 °C) to allow for the development of fungi, and then subcultures were made to obtain pure fungal cultures. Small wood segments from the wooden objects were also obtained for culturing. Two samples were obtained from each object and two sets of isolations were done. Sections were extracted from just beneath the wood surfaces, cut into minute segments, placed in different culture media outlined above, incubated at 24± 2° C and subcultures made to obtain pure cultures. Pure cultures from one set of isolations were identified using morphological characteristics and the second set was identified using molecular methods.

### DNA extractions, sequencing and identification of fungi

For fungi isolated from soil and wood in Egypt, taxonomic identification using morphological characteristics of fungal isolates was used based on the following keys: Pitt [32] for *Penicillium*; Raper and Fennell [33], Klich [34] for *Aspergillus*; Ellis [35, 36] for dematiaceous hyphomycetes; Booth [37] for *Fusarium*, Domsch *et al*. [38] for miscellaneous fungi, Rifai [39] for *Trichoderma* and Guarro *et al*. [40] for ascomycetes. For the second set of isolations, fungi obtained were brought to the University of Minnesota under USDA APHIS permit for DNA extraction and sequencing. All taxa from soils and wood were grown in pure culture on malt extract agar (15 g malt extract, 15 g agar and 1 L deinonized water). rDNA was extracted using a CTAB extraction procedure. Fungal hyphae from approximately ¼ of a Petri dish were scraped from the surface of an actively growing culture and suspended in 500 μl of cetyltrimethylammonium bromide (CTAB) lysis buffer and glass beads, vortexed for 1 min and centrifuged briefly to aggregate hyphal material. Supernatant was transferred to a new microcentrifuge tube and placed in a hot water bath (65°C) for 20 min. Following the hot water bath, 500 μl of chloroform/phenol/isoamyl alcohol (25:24:1) was added to each tube and mixed vigorously and centrifuged for 5 min. The supernatant was then removed and transferred to a clean microcentrifuge tube and isopropanol (stored at -20°C) was added (2/3 the amount of supernatant), gently mixed and incubated at room temperature for 5 min. Tubes were then centrifuged for 7 min and isopropanol was carefully removed. The DNA pellet remaining was washed with 500 μl of 70% ETOH (stored at -20°C) followed by centrifuging for 3 min after which the ETOH was removed and tubes were left in a sterilized bio-safety cabinet to air dry. DNA was rehydrated with 100 ml of sterile water. The internal transcribed spacer (ITS) region of rDNA was amplified using the primer combination ITS1F / ITS4 via PCR. One μl of DNA template was used in each PCR reaction that contained 1 μl of each primer (5 μM), 0.5 μl BSA, 12.5 μl of GoTaq Green Master Mix and 9.5 μl of sterile water in a thermocycler with the following program: 94°C for 5 min, 35 cycles of 94°C for 1 min, 50°C for 1 min, and 72°C for 1 min, followed by a final extension step of 72°C for 5 min. Amplicons were visualized by electrophoresis on a 1% agarose gel with a SYBR green prestain and transilluminated with a Dark Reader DR45 (Clare Chemical Research, Denver, Colorado). Sequencing was carried out using both forward and reverse primers using an ABI 3730xl DNA sequencer (Applied Biosystems, Foster City, CA, USA). A consensus sequence was assembled using Geneious 7.0 [41]. ITS sequences were obtained and the best BLAST match to GenBank sequences was determined using, if possible, accessions from taxonomic publications.

### Microscopic analyses

Sections from the wood samples were prepared for light microscopy and observations were made and photographs taken using Nikon-Eclipse E600 light microscope. Wood samples were prepared for scanning electron microscopy (SEM) using techniques described previously by Blanchette and Simpson [42]. Observations were made, and photographs taken using Hitachi S3500 scanning electron microscope.

## Results

**Fig 2:**
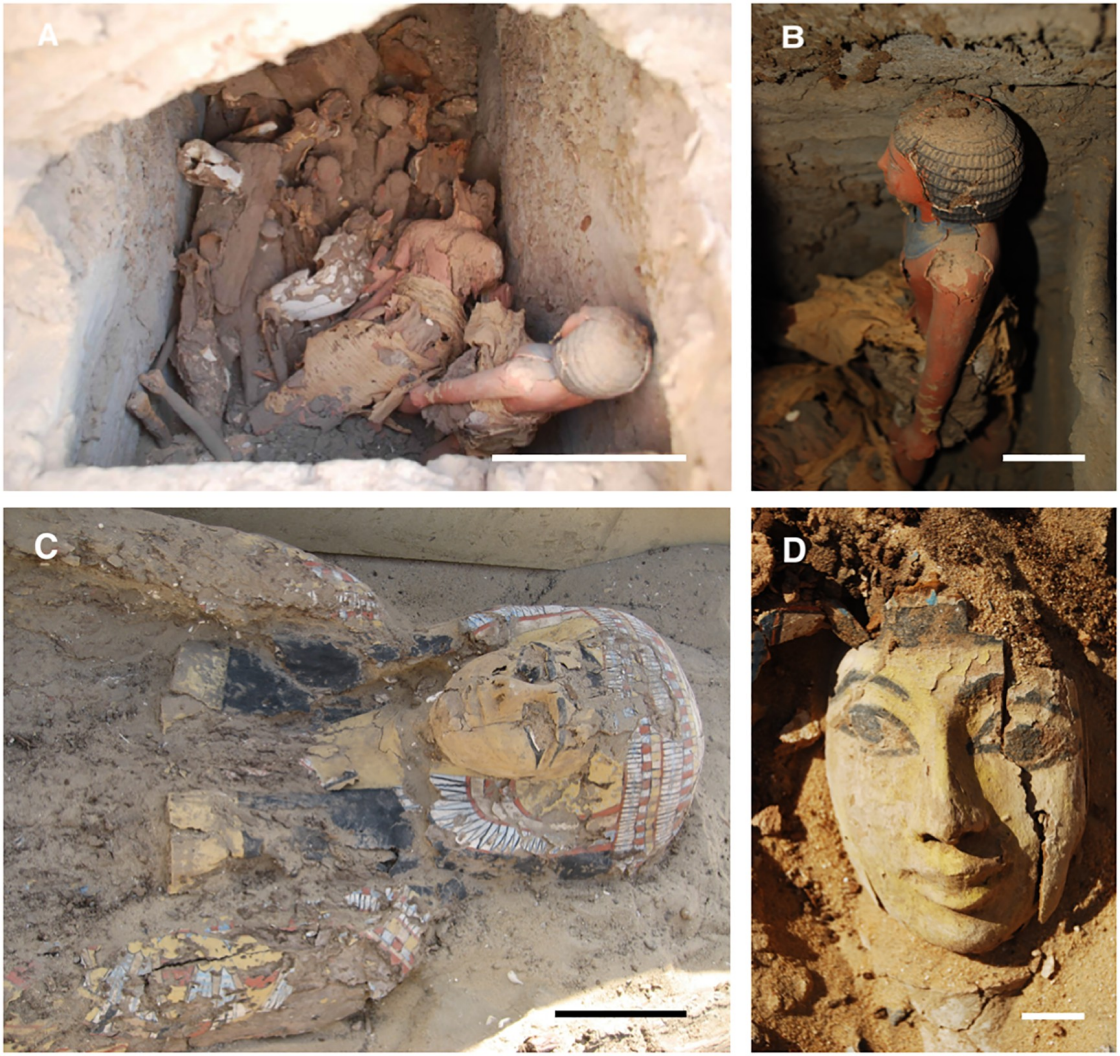
Wooden objects from the Middle Cemetery. A) Serdab chamber showing a variety of small wooden statues and other objects as they were found with severe degradation. B) Standing wooden statue as found in the tomb. C) Wooden coffin (AMC 2007, Unit 11, Feature 30, Level 59) showing the extensive degradation that had taken place. D) Coffin (AMC2013, Unit 26, Feature 26.9) showing condition of face at time of excavation. Photo A and B by Claudia Chemello and C and D by Suzanne Davis. Scale bar for A and C = 20 cm, B and D = 10 cm.

**Fig 3:**
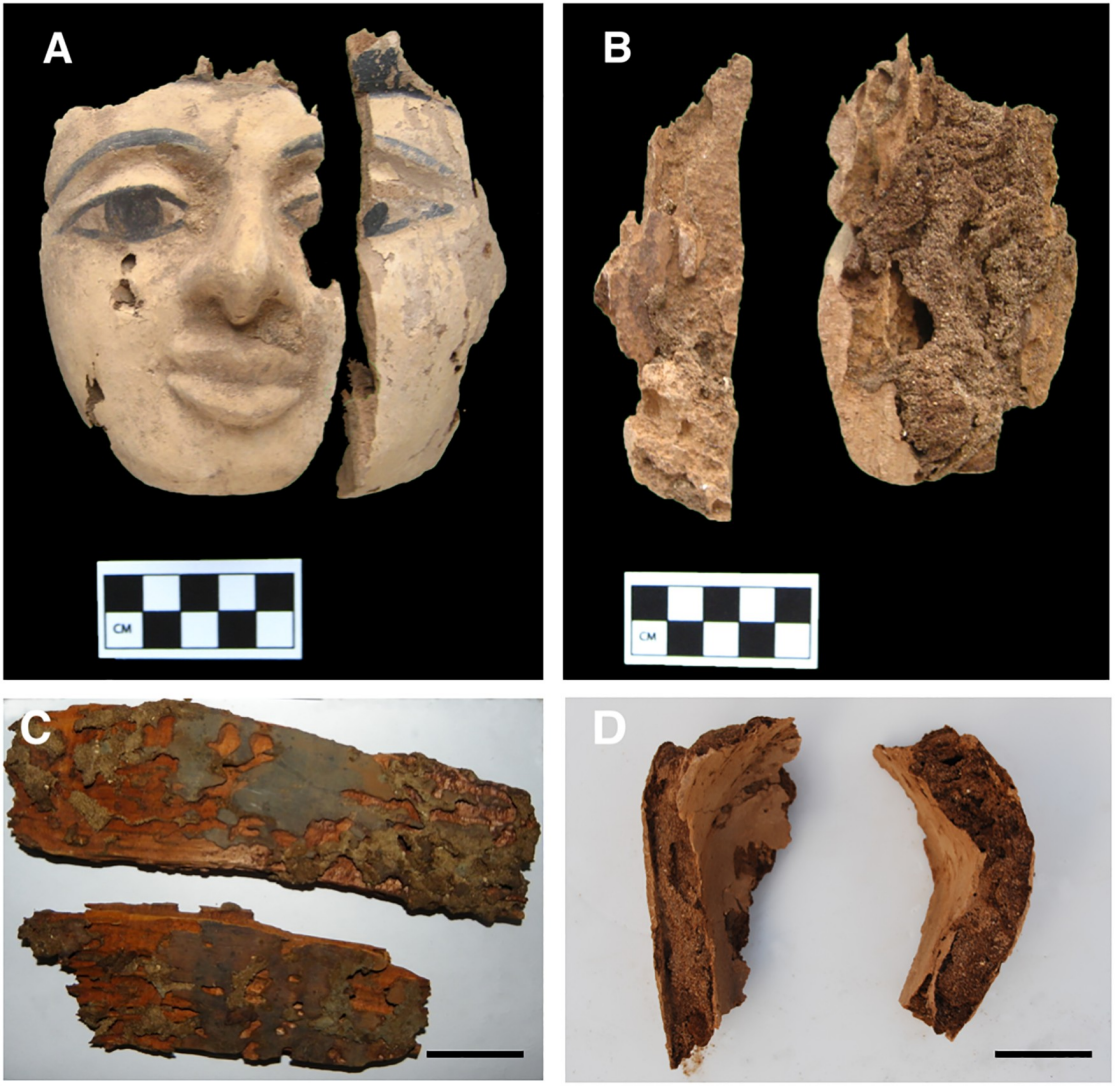
Wooden objects showing degradation. A) Front of coffin face mask (AMC2013, Unit 25, level 7) and B) reverse side of mask showing preserved surface and degraded interior with termite damage and wood degradation. C) Fragments of wooden planks (AMC1999 Unit 7E, level 2) with termite damage and wood decay. D) Degraded wood from coffin showing a thin outer surface remaining relatively intact but the inner zone consisted of only termite frass, sand particles and degraded wood. Scale bar = 5 cm.

**Fig 4:**
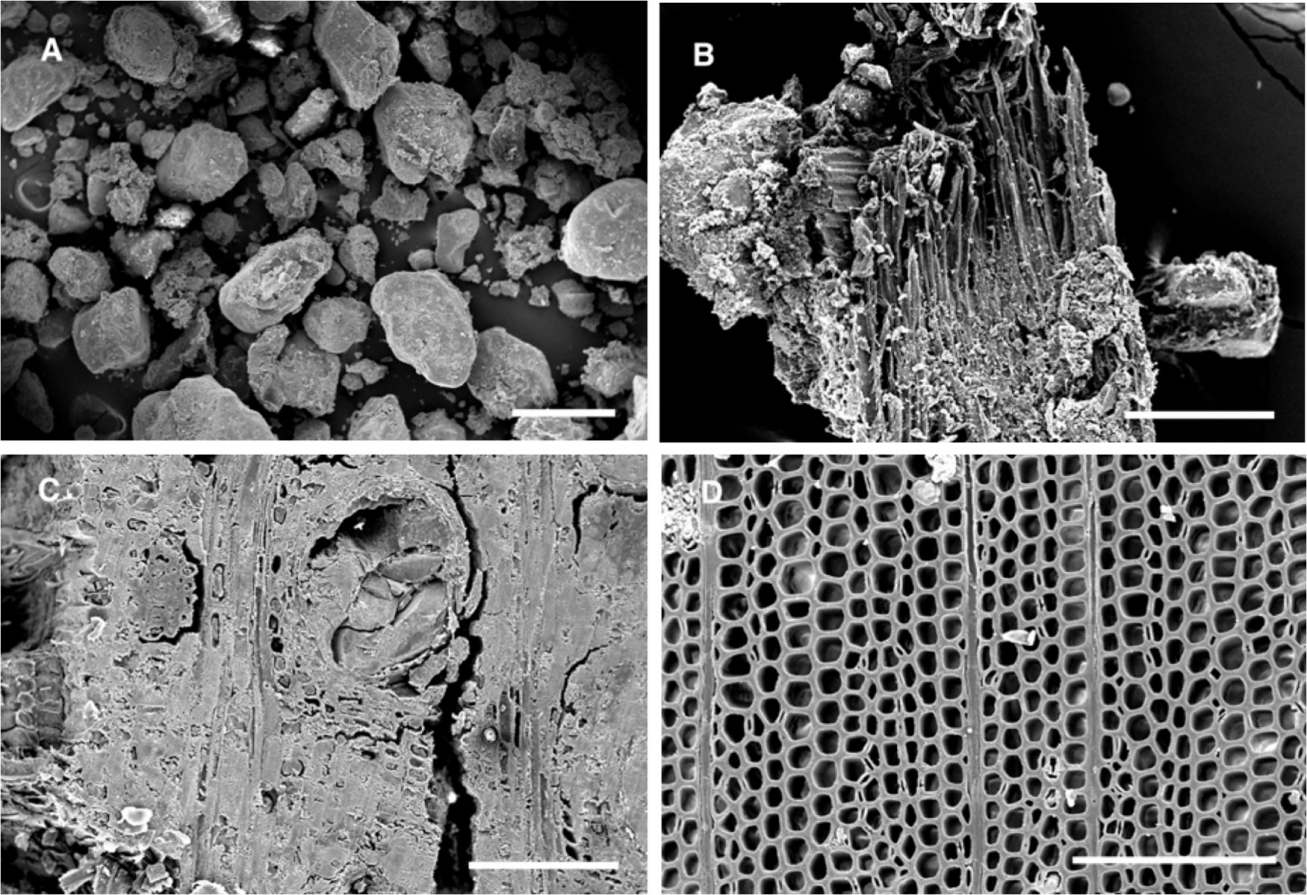
Scanning electron micrographs of termite damage and degraded wood from various objects excavated from the Middle Cemetery at Abydos. Termite frass (A) and small fragments of wood (B) were all that remained in some objects below the outer surfaces. Wood structure was preserved in some objects where resins, gesso or paint infiltrated the wood cells (C) as seen in this transverse section of wood showing cell lumina completely occluded. Wood preservation was also observed in some objects made from more decay and insect resistant woods such as *Cedrus* and *Juniperus* (D) as seen in this transverse section of wood showing relatively intact cells. Scale bar in A = 500μm and B, C, D = 200μm.

**Fig 5:**
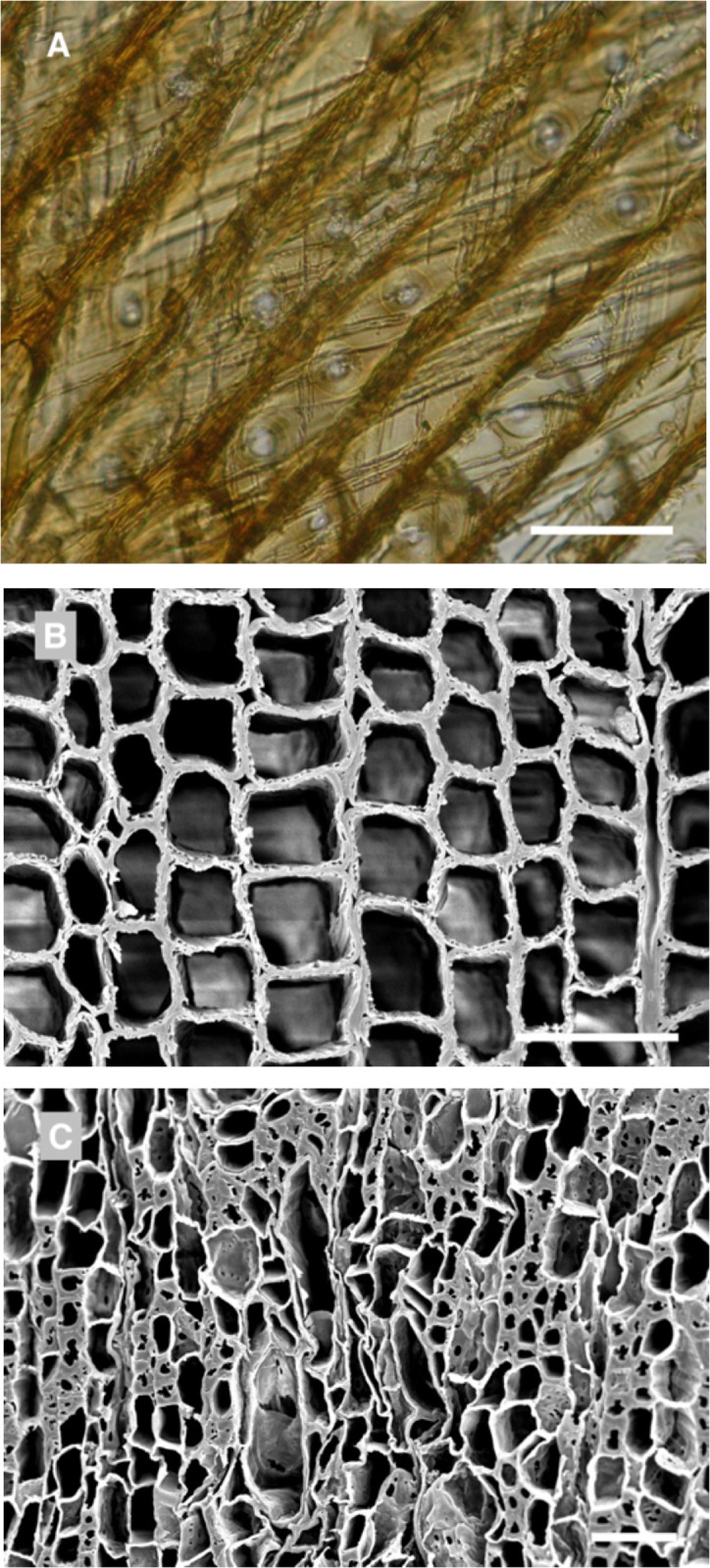
Micromorphology of decayed wood. Tangential section (A) and scanning electron micrographs of transverse sections (B and C) from wooden objects with wood degradation A) Type I soft rot in wood cells of *Cedrus* showing cavities that form inside the tracheid secondary walls. Soft rot cavities spiral within the cells. B) Small cavities are evident within the cell walls of the tracheids. C) An unknown hardwood with Type I soft rot and cavities within the secondary cell walls and also Type II soft rot causing an erosion of the entire secondary wall. Only a fragile middle lamella remains in some cells. Weakened sells appear distorted and some have collapsed. Scale bar = 50μm.

**Fig 6:**
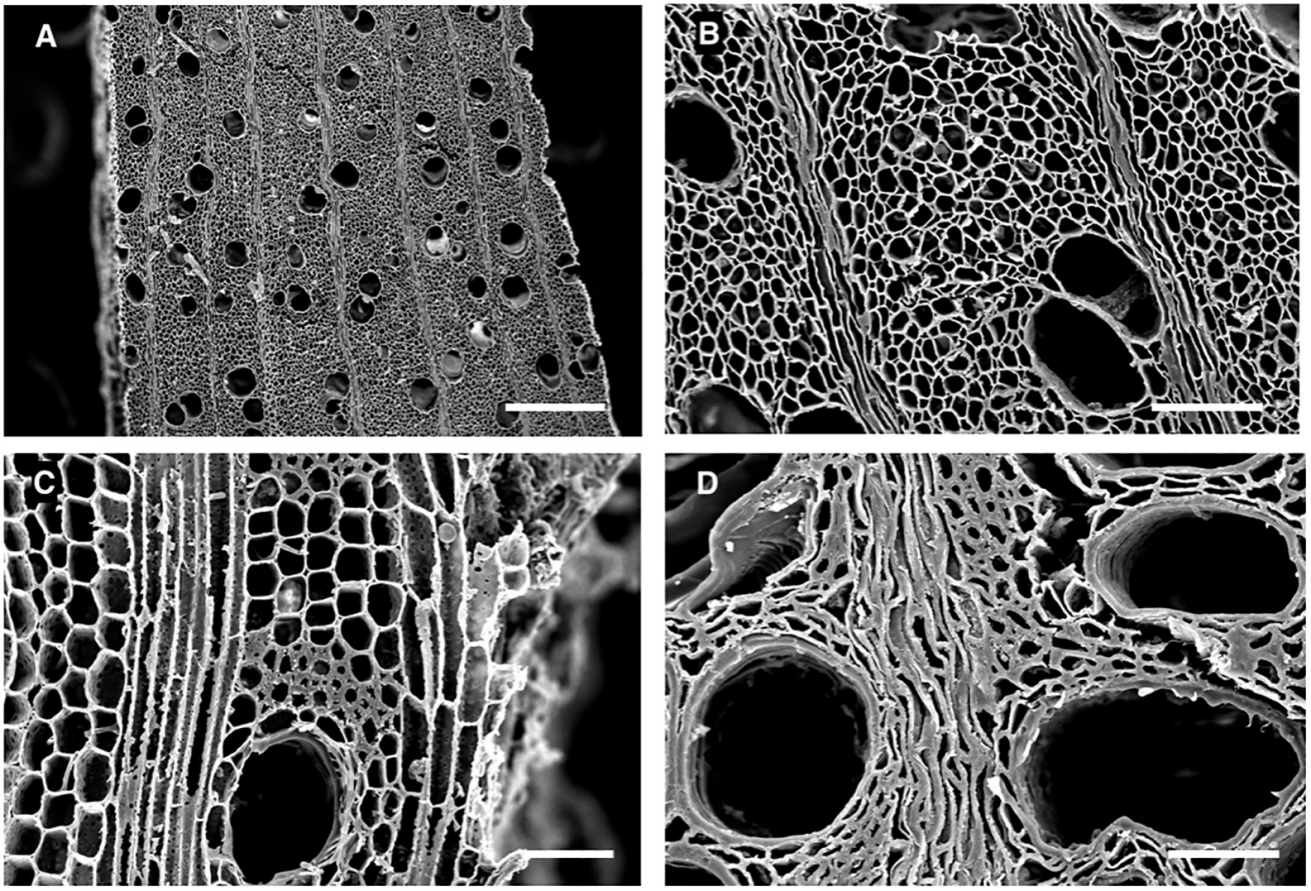
Scanning electron micrographs of transverse sections of degraded wood. A and B) Cell walls degraded by Type II soft rot from AMC 2013 field coffin sample 7. Secondary cell walls are degraded with most cells having only the middle lamella remaining. Although the wood structure can still be seen, the thin cells walls remaining are weak and fragile. C) Wood from coffin (AMC2013, Unit 28, Feat. 14, Burial 26.4) showing cells with Type II soft rot with degraded secondary walls and a thin middle lamella remaining as well as some cells with less severe attack that have some of the fiber secondary walls still present. D) Wood section from a box found in the Weni tomb (AMC01 level 1). The degraded cells walls caused by the soft rot have little strength and often break or collapse as seen in this micrograph. Scale bar in A = 250μ and B, C and D = 50μ.

Wooden objects excavated at Abydos were found to have severe deterioration and decay resulting in an exceedingly fragile condition (Figs 2 and 3). Small fragments of wood from the objects, used for microscopic observations of anatomical characteristics, were identified as *Cedrus*, *Juniperus*, *Acacia* as well as several other unidentified hardwoods. A combination of termite damage and wood decay was present. In some woods such as *Cedrus*, termite damage was near wood surfaces and large zones of unaffected wood remained (Fig 3). However, in many of the hardwood objects, there was extensive attack by termites and most of the wood below the surface had been converted to frass and small wood particles. Sand was also present that had been brought in with the termites. The surfaces of wood remained but lacked structural integrity. Micrographs of sections from areas with advanced decay showed termite frass, sand particles and segments of deteriorated wood (Fig 4). Sections from the surface of many of the wood samples showed a thin layer of cells that were completely occluded. These occlusions were from resin, gesso, paint or other substances that had infiltrated the wood surfaces (Fig 4). Relatively sound cell walls were also found in some areas of *Cedrus* (Fig 4) but in other zones, soft rot attack by wood decay fungi was present (Fig 4). Light microscopy (Fig 5) of wood sections revealed cavities within the secondary wall of tracheids that was characteristic of Type I soft rot. Advanced stages of decay were present and many soft rot cavities were seen in a spiral pattern within the secondary walls. In transverse sections, small cavities were seen within tracheids (Fig 5). Wood from objects that had been made of various hardwoods also had evidence of soft rot. Some cavities within secondary walls were present but more commonly observed was a Type II soft rot where the secondary cell walls were eroded (Fig 5). In some cells with advanced decay, the entire secondary cell wall was removed leaving only the middle lamella. The altered cells were often collapsed and distorted. Sections from other wooden objects showed similar patterns of degradation with Type II soft rot in objects made from hardwood (Fig 6). The secondary walls were degraded leaving only a weak and fragile framework of the middle lamella and cell walls were often fractured and collapsed (Fig 6).

The texture of soils that were sampled from the excavation site were clay loam to loamy fine sand according to particle size distributions as described by United States Department of Agriculture [30] and calculated online by the soil texture calculator (https://www.nrcs.usda.gov/wps/portal/nrcs/detail/soils/survey/?cid=nrcs142p2_054167). The soil samples were slightly alkaline with soil pH values ranging from 7.5 and 7.8. Electric conductivity (EC) ranged between 207 and 1508 microSiemens/cm. The mean values of percent organic matter ranged between 1.4 and 4.96%. Soils were moderately saline with salinity ranging from 132 to 965 ppm (Table 2). Values for electrical conductivity for four out of the five samples, which also provides an assessment of soil salinity, was very high.

**Table 2:** Physicochemical parameters of soil samples collected adjacent to where wood was excavated.

Soil Sample	pH	% Organic Matter	Electric Conductivity (micro-Siemens/cm)	Salinity (ppm)	% Sand	% Silt	% Clay
1	7.86±0.07	1.48±0.02	1070.4 ±4.41	685.06 ±0.17	74.74	21.16	4.11
2	7.61±0.07	2.25±0.01	1452.2 ±3.69	929.41 ±0.15	55.09	35.33	9.58
3	7.80±0.04	2.41±0.02	1508.4 ±3.43	965.38 ±0.22	71.93	14.39	13.68
4	7.85±0.02	2.75±0.02	1143.6 ±2.34	731.90 ±0.30	77.54	18.35	4.11
5	7.53±0.0	4.96±0.06	207.6 ±0.02	132.86 ±0.15	36.84	28.95	34.21

Nine different taxa of fungi were identified from soil samples and 16 from wood. All identified taxa were deposited in the culture collection at the Suez Canal University Fungarium (SCUF) of Arab Society for Fungal Conservation, Botany Department, Faculty of Science, University of Suez Canal, Egypt. The prevailing genera in soils were *Aspergillus* species (5 species; 55.55% of the total isolates), and the remaining taxa were represented only by one species each. Based on recovered total colony forming units (CFU), site number 5 had the highest number (400000 CFU), while site number 1 showed the lowest count (1200 CFU). The fungal taxa found in soil samples were similar to those found in wood and *Aspergillus* species were the most numerous taxa found in wood samples. In addition to *Aspergillus*, other taxa identified by morphological characteristics of fungal cultures or sequencing the ITS region of rDNA included species of *Chaetomium*, *Cladosporium*, *Fusarium*, *Penicillium*, *Stemphylium* and *Talaromyces*, *Trichoderma* (Tables 3, 4 and 5).

**Table 3:** Total count (TC) of colonies/g dry soil, number of cases of isolation (NCI) from 5 sites, percentage frequency and presence or absence within sites of fungal taxa recovered on isolation media at 28°C.

Taxa	TC	NCI	% F	Presence/Absence				
				S1	S2	S3	S4	S5
*Aspergillus flavus*	31400	40	80	+	+	+	+	+
*Aspergillus fumigatus*	20900	20	40	-	+	+	+	+
*Aspergillus niger*	251000	44	88	+	+	-	+	+
*Aspergillus ochraceus*	60600	27	54	-	+	-	+	+
*Aspergillus terreus*	20000	15	30	-	-	-	-	+
*Cladosporium cladosporioides*	300	10	20	+	+	-	+	-
*Fusarium oxysporum*	5700	17	34	+	+	+	-	-
*Stemphylium globuliferum*	100	8	16	+	-	-	-	-
*Trichoderma longibrachiatum*	20400	16	32	+	+	+	-	+

**Table 4:** Taxa of fungi identified using morphological characteristics from ancient wood samples after growing on media at 28°C.

Wood Sample #	Taxa
2–1	*Aspergillus flavus*
2–2	*Trichoderma* sp.
3–1	*Aspergillus flavus*
4–1	*Trichoderma* sp.
5–1	*Trichoderma* sp.
6–1	*Chaetomium globosum*
6–2	*Aspergillus terreus*
6–3	*Trichoderma* sp.
7–2	*Aspergillus flavus*
7–4	*Penicillium* sp.
8–1	*Aspergillus fumigatus*
9–1	*Aspergillus fumigatus*
10–1	*Aspergillus fumigatus*
11–1	*Aspergillus flavus*
13–1	*Aspergillus fumigatus*
14–2	*Aspergillus fumigatus*
15–1	*Aspergillus fumigatus*
17–1	*Aspergillus fumigatus*
18–2	*Aspergillus fumigatus*
19–1	*Aspergillus fumigatus*
20–1	*Aspergillus niger*
20–2	*Aspergillus flavus*
21–1	*Aspergillus ochraceous*
21–2	*Penicillium* sp.
21–3	*Aspergillus niger*
26–1	*Aspergillus niger*
27–1	*Aspergillus flavus*
28–1	*Aspergillus niger*
28–2	*Aspergillus fumigatus*
28–3	*Penicillium* sp.
29–1	*Aspergillus fumigatus*
33–1	*Aspergillus ochraceous*
33–2	*Penicillium* sp.
33–3	*Aspergillus flavus*
34–1	*Aspergillus fumigatus*
34–2	*Aspergillus niger*
34–3	*Aspergillus ochraceous*

**Table 5:** Taxa identified from fungi isolated from wood by Best Blast Match of the ITS region of rDNA.

Isolate #	Best BLAST Match	% Identity	GenBank Access. #
**Abydos Wood**			
3–1	*Aspergillus flavus var*. *oryzae*	100%	MK095969
3–2	*Aspergillus flavus var*. *oryzae*	100%	MK095970
3–3	*Aspergillus terreus*	99%	MK095983
4–3	*Aspergillusflavus var*. *oryzae*	100%	MK095971
5–1	*Trichoderma longibrachiatum*	100%	MK095972
5–2	*Talaromyces liani*	99%	MK095973
5–8	*Fusarium oxysporum*	100%	MK095974
6–1	*Aspergillus niger*	100%	MK095975
6–2	*Aspergillus terreus*	100%	MK095976
11–2	*Aspergillus flavus*	100%	MK095977
13–1	*Aspergillus fumigatus*	100%	MK095978
13–2	*Aspergillus fumigatus*	100%	MK095979
25–1	*Penicillium pinophilum*	100%	MK095980
25–2	*Cladosporium cladosporioides*	100%	MK095984
25–3	*Stemphylium vesicarium*	99%	MK095985
25–4	*Cladosporium cladosporioides*	99%	MK095986
25–5	*Cladosporium anthropophilum*	99%	MK095987
25–6	*Stemphylium paludiscirpi*	99%	MK095988
25–7	*Aspergillus niger*	99%	MK095989
29–1	*Stemphylium vesicarium*	99%	MK095990
29–2	*Cladosporium limoniforme*	99%	MK095991
**Abydos Soil**			
S3-2	*Fusarium oxysporum*	100%	MK095992
S3-3	*Fusarium oxysporum*	100%	MK095993
S5-1	*Aspergillus flavus*	100%	MK095994

## Discussion

The rate and extent of wood decomposition by fungi is governed by environmental factors but even in the most extreme environments degradation can take place. Moisture is essential for decomposition processes and wood cell walls must have sufficient moisture to be above the fiber saturation level in order for extracellular microbial enzymes and non-enzymatic processes to cause degradation [43]. The cemetery at Abydos is a dry desert site with annual rainfall of about 1mm but greater amounts of rain can occur infrequently [44]. Average humidity is approximately 60% and this decreases in the winter months to less than 30%. Temperatures can range from 2° C in February to 45° C during June. Although these arid conditions would appear to restrict wood decay from occurring, flooding of the Nile and increases in the water table in soils beneath Abydos apparently contributes sufficient moisture intermittently for below ground microbial activity and degradation to take place [45].

Soil characteristics indicate an elevated pH and the presence of salts in the Abydos soil. Electrical conductivity and soil salinity were moderately high and most soil samples (four out of five) had high levels of salts. Often salts accumulate in desert soils from the rise and fall of ground water and precipitation of salts during evaporation as well as accumulations entering by wind. These environmental conditions favor soft rot fungi that have a higher tolerance for adverse conditions such as high pH and restricts decay by basidiomycetes that cause white and brown rot and prefer lower pH and non-saline conditions [10–13].

The fungi that were isolated are taxa that are common to desert regions and have been reported previously from Egypt [46–49]. A large amount of diversity in taxa was found in the two sets of wood samples that were used for isolations. Molecular methods provided identification to a species level based on the best blast match to accessions in Gen Bank from a recent taxonomic treatise for each specific group of fungi isolated from wood samples. These identifications are based on the ITS region and for some taxa with large amounts of diverse species such as *Aspergillus*, using only the ITS region is not sufficient to separate all species. Additional molecular analyses using other genes may be needed to confirm species identity.

Wood degradation has been reported and considered a problem over the past many decades during excavations of archaeological wood in Egypt including the excavation of the Tomb of Tutankhamen. In 1923, Carter reported “the existence of past damp in the tomb… that nourished fungoid growth” [50]. Moisture in the Tutankhamen tomb that facilitated the degradation process was thought to occur from intermittent rain storms that allowed water to filter down into the tomb chambers [50]. The poor condition of the wood and other artifacts found in the tomb resulted in great concern and it took 10 years to remove the funerary objects from the Tutankhamen tomb. Although it has been almost 100 years since the Tutankhamen excavations, we still have incomplete information on degradation processes occurring in these desert sites and knowledge of the fungi responsible for the degradation is very limited. Investigations of wooden objects from ancient Egypt in museums have revealed a number of different types of biological degradation and non-biological deterioration processes that have affected the various woods [16–18]. These include wood decay from various types of fungi including brown and soft rot, as well as a chemical corrosion of wood by salts. Other studies of wood degradation in extreme environments have shown that soft rot fungi may be the primary cause of wood decay and degradation by these fungi can take place under conditions that exclude other more aggressive fungi usually found in more conducive environments.

Decay in the Abydos wood had distinct characteristics of Type I and Type II soft rot (Figs 2 and 3). Type I soft rot causes cavities to form within the secondary walls of the wood whereas Type II soft rot can erode the secondary wall and in advanced stages, the entire secondary wall may be completed removed leaving only the middle lamella between cells. Wood with advanced stages of decay is exceedingly fragile and the residual wood can break with the slightest movement or crushed with a small amount of pressure. Several of the fungal species that were isolated from the Abydos wood are known to cause a soft rot type of wood decay [51, 52]. The isolations completed in the study present here provide information on the fungi currently in the wood and may not reflect the taxa responsible for the attack that may have taken place hundreds of years ago. However, they do reflect the dominant fungi at this site and the current fungi that are alive and can be isolated from the wooden objects. Although the degradation may have taken place hundreds or even thousands of years ago, the result obtained provide insight about the type of fungi that were the likely cause of the degradation. In a recent study of fungi associated with archaeological wood from museums in Egypt, the taxa found were similar to those in our study with *Aspergillus* species being among the most dominant [24]. Many of the fungi we report in Tables 3, 4 and 5 are adapted to arid conditions, elevated pH and high salinity. Similar taxa were found in soils adjacent to the buried woods and suggest that these soil fungi are opportunistic organisms that can attack wood or other carbon sources that are placed in the soil. Wood decay by soft rot fungi is slow to progress but studies have shown in arid buried sites having high pH as well as other sites with extreme environments, such as coastal sites in Antarctica, that these fungi cause degradation whenever the conditions are suitable [10, 11, 13]. The fungi may go dormant when moisture or other environmental conditions are limiting and can reactivate as conditions change. Decay can continue a slow progression over hundred or even thousands of years as demonstrated in wood from Tumulus MM in Turkey thought to be the burial site for King Midas [13, 42]. Nitrogen is often also a limiting factor during the degradation of wood and soft rot fungi have been shown to be able to recycle nitrogen and translocate it to the advancing front of the mycelium. This allows small amounts of nitrogen to be continually reutilized as the decay fungus continues to move throughout the wood over exceptionally long periods of time [13]. The fungi isolated and identified in our study provide important information on the fungi that are active at Abydos and reveals the many species that can be found under these environmental conditions that can cause soft rot.

Eight species of subterranean termites have been reported in Egypt including four species of *Psammotermes* [53]. These termites are abundant in arid regions of Upper Egypt and they attack any substrate containing cellulose. Termites can be a serious problem in Egypt attacking buildings and other wood in the environment, grain and even straw used in mud bricks. Archaeological wood can also be affected if termites can gain access to it. In Abydos, although the wood was buried and often placed in mud brick structures, termites found and attacked the wooden objects resulting in extensive damage. The residual wood that remained often consisted of just the wood surfaces or wood adjacent to the surface cells that were coated with gesso paint or resin. Some wood, such as *Cedrus*, has naturally occurring heartwood compounds that resist decay, and some objects made from this wood resisted attack with more wood surviving. An investigation of the fungi associated with the sand termite, *Psammotermes hypostoma*, in Egypt showed many similar species of fungi to those we isolated in this study including *Aspergillus*, *Cladosporium*, *Fusarium* and *Trichoderma*. These fungi appear common to arid desert regions and may be widely distributed in soils and on insects such as termites. Many of these fungi can colonize wood causing some biomass loss in laboratory studies and produce decay that is a Type I or Type II soft rot [51, 52]. Since our study showed that soft rot was associated with termite damage in all the wooden objects examined more investigation appears warranted to better understand the association of these fungi with termites.

The extensive attack by termites and decay by soft rot fungi in buried wood at Abydos pose serious problems during excavation. The lack of structural integrity of the wood due to degradation can result in disintegration of the object into dust like particles if not treated properly during excavation. Preliminary studies of the Abydos wood revealed that surface treatments in-situ can be used to stabilize pigments and the surfaces of the wooden objects. However, to lift large sections of the wood for transport to the conservation laboratory, additional support is needed. To provide this additional support an *in-situ* treatment with a hydrocarbon wax, cyclododecane, was used [54]. This solidified around the object allowing excavation to take place. Once in the laboratory, the cyclododecane was allowed to sublime and surfaces were cleaned, and wood consolidated with B72 acrylic resin and other consolidants. Additional details of the conservation methods have been reported [3]. Although conservation treatment in small case-studies has been successful, many questions about the best conservation method for these woods remain. The research reported here provides an important and essential first step in understanding the condition of ancient wood being excavated from Abydos and can also be useful to studies of deterioration and decay occurring in archaeological wood from other arid regions. The results also reveal the identity of the fungi that are still viable within the wooden objects. Additional research is needed to identify the most successful procedures for consolidating termite frass, sand and decayed wood fragments within objects, filling voids that have developed in decayed wood; and preserving fragile wood surfaces and adhering them to the degraded interior substrates of wood, frass and sand particles.

## References

[1] O’Connor D. Abydos: Egypt’s First Pharaohs and the Cult of Osiris. London: Thames and Hudson 2009.

[2] Landvatter T. 2013. Burial practices and ritual landscapes at Ptolemaic Abydos: The 2011 and 2012 seasons of the Abydos Middle Cemetery. Near Eastern Archaeology 2013;76(4):235–45.

[3] Davis SL, Chemello C. CSI Abydos: Conservation and scientific investigation of wood Funerary artifacts at the Abydos Middle Cemetery. Bulletin of the American Research Center in Egypt 2014;204:13–20.

[4] Richards JE. Text and context in Late Old Kingdom Egypt: The Archaeology and historiography of Weni the Elder. Journal of the American Research Center in Egypt 2002;39:75 102.

[5] Richards JE. Society and death in ancient Egypt: Mortuary landscapes of the Middle Kingdom. Cambridge: Cambridge University Press 2005.

[6] Richards JE. The archaeology of excavations and the role of context In Hawass Z, Richards J. The archaeology and art of ancient Egypt: Essays in honor of David B. O’Connor, Cairo: Supreme Council of Antiques 2007 p. 313–19.

[7] Richards JE. Herbich T. The Loss and rediscovery of the Vizier Iuu at Abydos: Magnetic survey in the Middle Cemetery In Czerny E, editor. Festschrift Manfred Bietak. Vienna: Denkschriften der Gesamtakademie 2005 p. 141–49.

[8] Blanchette RA. A review of microbial deterioration found in archeological wood from different environments. International Biodeterioration and Biodegradation 2000;46:189–204.

[9] Blanchette RA, Nilsson T, Daniel G, Abad A R. In Rowell RM, Barbour R J, editors. Archeological Wood: Properties, Chemistry, and Preservation. Washington DC: American Chemical Society; 1990 p. 141–74.

[10] Blanchette RA, Held BW, Jurgens JA, Haight JE.Wood deterioration in Chacoan great houses of the southwestern United States. Conservation and Management of Archaeological Sites 2004;6:204–12.

[11] Blanchette RA, Held BW, Jurgens JA, McNew DL, Harrington TC, Duncan SM, et al Wood destroying soft rot fungi in the historic expedition huts of Antarctica. Applied and Environmental Microbiology 2004;70:1328–35. 10.1128/AEM.70.3.1328-1335.2004 15006750 PMC368374

[12] Blanchette RA, Held BW, Arenz BE, Jurgens JA, Baltes NJ, Duncan SM, et al An Antarctic hot spot for fungi at Shackleton’s historic hut on Cape Royds. Microbial Ecology 2010;60:29–38. 10.1007/s00248-010-9664-z 20386896

[13] Filley T, Blanchette RA, Simpson E, Fogel M. Nitrogen cycling by wood decomposing soft-rot fungi in the ‘King Midas tomb’ Gordion, Turkey. Proceedings of the National Academy of Science USA 2001;98:13346–13350. 10.1073/pnas.221299598 PMC60873 11606731

[14] Oritz R, Parraga M, Navarrete J, Carrasco I, de la Vega E, Ortiz M, et al Investigations of Biodeterioration by Fungi in Historic Wooden Churches of Chiloe, Chile. Microbial Ecology 2014;67:568–575. 10.1007/s00248-013-0358-1 24407313

[15] Zaremski A, Palanti S, Mannucci M, Gastonguay L, Le Floch G. Molecular diagnosis by PCR-DHPLC technique of wood-decay fungi in historical buildings in Italy. Pro Ligno 2011;7(4):92–7.

[16] Blanchette RA, Haight JE, Koestler RJ, Hatchfield, PB, Arnold, D. 1994. Assessment of deterioration in archaeological wood from Egypt. Journal of the American Institute of Conservation 1994;33:55–70.

[17] El-Hadidi NMN. Decay of softwood in archaeological artifacts. Studies in Conservation 2017;62:83–95.

[18] Nilsson T, Daniel G. Structure and the aging process of dry archaeological wood In Rowell RM, Barbour RJ editors. Archeological Wood: Properties, Chemistry, and Preservation Washington DC: American Chemical Society 1990 p 67–86.

[19] Darwish SS, El Hadidi NMN, Mansour M. The effect of fungal decay on *Ficus* sycomorus wood. International Journal of Conservation Science 2013;4(3):271–82.

[20] El-Fouly MZ, Shahin AM, El-Bialy HA. Biological control of sapstain fungi in Egyptian wood stores and infected trees. Annals of Microbiology 2011;61:789–99.

[21] Hamed SAM. In-vitro studies on wood degradation in soil by soft-rot Fungi: *Aspergillus niger* and *Penicillium chrysogenum*. International Biodeterioration and Biodegradation 2013; 73:98–102.

[22] Mansour M. Studies on protection and preservation of wood archaeological furniture against mould fungi, PhD dissertation, Cairo Egypt: Cairo University. 2007.

[23] Mansour M, Ahmed H. Occurrence of fungi on some deteriorated ancient Egyptian materials and their controlling by ecofriendly products. Egyptian Journal of Archaeological and Restoration Studies 2012; 2(2):91–101.

[24] Osman ME, El Shaphy AAR, Meligy DA, Ayid MM. Survey for fungal decaying archaeological wood and their enzymatic activity. International Journal of Conservation Science 2014;5:295–308.

[25] Blanchette RA, Held BW, Hellmann L, Millman L, Büntgen U. Arctic driftwood reveals unexpectedly rich fungal diversity. Fungal Ecology 2016;23:58–65.

[26] Held BW, Blanchette RA. Deception Island Antarctica harbors a diverse assemblage of wood decay fungi. Fungal Biology 2017;121:145–57. 10.1016/j.funbio.2016.11.009 28089046

[27] Loyd AL, Barnes CW, Held BW, Schink MJ, Smith ME, Smith JA, et al Elucidating "lucidum": Distinguishing the diverse laccate Ganoderma species of the United States. PLOS ONE 2018; 10.1371/journal.pone.0199738 PMC6051579 30020945

[28] Allen S, Grimshaw HM, Parkinson JA, Quarmby C, Roberts JD. Chemical analysis In: Chapman SB editor. Methods in plant ecology. Oxford: Blackwell Scientific; 1976 p. 411–60.

[29] Wilde SA, Weight GK, Ixer JG. Soil and plant analysis for tree culture. New Delhi, Oxford: B.H. Publication Company 1972.

[30] United States Department of Agriculture. Particle size analyses. In: Soil Service Laboratory Manual 42 Version 3. Washington DC: USDA. National Soil Survey Center. 1996.

[31] Garrett SD. Soil fungi and soil fertility. 2nd Ed, Oxford, New York: The Macmillan Company 1981.

[32] Pitt JI. The genus *Penicillium* and its teleomorphic states *Eupenicillium* and *Talaromyces*. London: Academic 1980.

[33] Raper KB, Fennell DI. 1965 The genus *Aspergillus*. Baltimore: Williams & Wilkins.

[34] Klich MA. Identification of common *Aspergillus* species. Utrecht, Netherlands: Centralbureau voor Schimmelcultures 2002

[35] Ellis MB. Dematiaceous hyphomycetes, Kew, Surrey, England: Commonwealth Mycological Institute 1971.

[36] Ellis MB. More Dematiaceous hyphomycetes, Kew, Surrey, England: Commonwealth Mycological Institute 1976.

[37] Booth C. *Fusarium*, Laboratory Guide to the Identification of the Major Species. Kew, Surrey, England: Commonwealth Mycological Institute 1971.

[38] Domsch KH, Gams W, Anderson TH. Compendium of soil fungi. Eching, Germany: IHW-Verlag 2007.

[39] Rifai MA. A revision of the genus Trichoderma. Mycological Papers 1969;116:1–56.

[40] Guarro J, Gene J, Stchigel AM, Figueras MJ. Atlas of soil ascomycetes CBS Biodiversity Series 10, Holland: Centralbureau voor Schimmelcultures 2012.

[41] Kearse M, Moir R, Wilson A, Stones-Havas S, Cheung M, Sturrock S, et al Geneious basic: an integrated and extendable desktop software platform for the organization and analysis of sequence data. *Bioinformatics* 2012;28: 1647–1649. 10.1093/bioinformatics/bts199 22543367 PMC3371832

[42] Blanchette RA, Simpson E. Soft rot decay and wood pseudomorphs in an ancient coffin (700 BC) from tumulus MM at Gordion, Turkey. International Association of Wood Anatomists Bulletin 1992;13:201–13.

[43] Eriksson K-E, Blanchette RA, Ander P. Microbial and enzymatic degradation of wood and wood components. Berlin:Springer 1990.

[44] Diab MS, El-Shayeb MH, Abdel-Moneim AA, Said MM, Zaki SR. Evaluation of water resources and land suitability for development in the southern part of Sohag, Upper Egypt. Annals of the Geological Survey of Egypt 2002;XXV:487–512.

[45] Sefelnasr A, Abdel-Moniem A, Abu El-Magd S. Groundwater level-rise monitoring and recharge determination at an old archaeological site: Abydos, Sohag, Egypt. Eighth International Conference on the Geology of Africa. 2015. p iv- 69 –iv- 83. Assiut, Egypt.

[46] Abdel-Azeem AM. Effect of overgrazing on vegetation, microbes and soil in Ismailia-desert habitat In: Pineda FD, Casado MA, deMiguel JM, Montalvo J, editors. Diversidad Biológica / Biological Diversity. Madrid: Centro de Estudios Ramón Areces S.A; 1991 p 241–46.

[47] Abdel-Azeem AM. Ecological and taxonomical studies on ascospore producing fungi in Egypt. Ph.D Thesis, Faculty of Science, Suez Canal University, Egypt; 2003.

[48] Abdel-Azeem AM. Operation Wallacea in Egypt. I- A preliminary study on diversity of fungi in the world heritage site of Saint Katherine, Egypt. Assiut University Journal of Botany 2009;38(1): 29–54.

[49] Abdel-Azeem AM, Ibrahim ME. Diversity of terrophilous mycobiota of Sinai. Egyptian Journal of Biology 2004;6:21–31.

[50] Carter H. The Tomb of Tutankhamen: Discovered by the late Earl of Carnarvon and Howard Carter. Vol. 3 London: Cassell and Company LTD 1923.

[51] Hamed SAM. In-vitro studies on wood degradation in soil by soft-rot fungi: Aspergillus niger and Penicillium chrysogenum. International Biodeterioration and Biodegradation 2013;78:98–102.

[52] Nilsson T. Studies on wood degradation and cellulolytic activity of microfungi. Studia Forestalia Suecica Nr. 104 1973.

[53] Moharram AM, Bagy MMK, Abdel-Galil FA. Fungi associated with the sand termite Psammotermes hypostoma in Assiut, Egypt. Mycologia 192;84:930–35.

[54] Balachandran, S. The Use of cyclododecane in field stabilization and storage of archaeological finds. In: Williams E, Pechy C, editors. The Conservation of archaeological materials. Current trends and future directions. British Archaeological Reports International Series 2116 Oxford: Archaeopress 77–87; 2010.

